# The History and Capabilities of Herbal Simples

**Published:** 1890-06-28

**Authors:** W. T. Fernie


### The History and Capabilities of Herbal Simples.

By W. T. Fernie, M.D.

*(Continued from page 148.)*

### XII.—BUCKTHORN.

"O! DURA ILIA MESSORUM!"—Ye gods! what strong stomachs the reapers must own!—So sang Horace and Sir Theodore Martin in the third Epode. And the same thing might have been said with equal force of English rustics until late in the present century, who, when requiring an aperient dose for themselves or their children, had recourse to the buckthorn. This—the Rhamus Catharticus—was formerly in common use as a purgative simple, but its action was so violent and attended with such gripings that as time went on it was discarded, and it is now employed in this respect almost exclusively for cattle and animals. Dodaeus taught about buckthorn berries—they be not meet to be ministered but to young and lusty people of the country which do set more store of their money than their lives. The shrub is frequent in our woods and hedges, growing near brooks, and chiefly on chalk. It is named buckthorn from the German buxdorn, boxthorn, and is called also waythorn, rainberry thorn, and rhineberries. The ripe berries are of a bluish black colour, and each contains four seeds. The flesh of birds which eat these berries is said to be purgative. When the juice is given medically it causes severe stomachache, with much dryness of the throat. For this reason Sydenham always ordered a basin of soup after it. The colouring matter of the berries when mixed with alum makes the pigment known as sap-green. Chemically the active principle of the buckthorn is Rhamno-Cathartine. In the United States the shrub is largely used for making hedges. A milder kind of buckthorn also grows freely in England, the Rhamnus frangula, or so called black berry-bearing alder, but this appellation is a mistake, because botanically the alder is never berry-bearing. This black buckthorn is a slender shrub, which grows in our woods and thickets. The juice of its berries is aperient without being irritating, and is well suited for persons of delicate constitution. It possesses the merit of continuing to answer in smaller doses after the patient has become habituated to its use. The berry of the Rhamnus frangula may be known by its containing only two seeds. Country people give the bark boiled in ale for jaundice, and as a purgative. This bark is the black dogwood of gunpowder makers. Lately a certain medicine for habitual constipation has become highly popular with medical men in this country which is known as Cascara Sagrada. This is really an American buckthorn, the Rhamnus Pursiana, and it possesses no true advantage over our black alder buckthorn, but the bark of this latter must be used a year old or it also will cause griping. A fluid extract is made of it by our leading druggists, of which from half to one teaspoonful may be given as a dose; this is likewise a tonic to the intestines, and is especially valuable in cases of piles. Lozenges of alder buckthorn are sold under the name of aperient fruit lozenges; one, or perhaps two may be taken as a dose. Syrup of buckthorn was the old-fashioned medicine when the Rhamnus Catharticus used to be employed, but this is now quite out of date for the human subject. The shrub has spinous, thorny branchlets, and later herbalists supposed the name rhamnus to have been given because it is set with thorns like as the ram. At one time the buckthorn was a puzzle even to Royalty, as we may judge from the following lines respecting it:—

> "Hicum, peridicum—all clothed in green;
> The king could not tell it, no more could the queen:
> So they sent to consult wise men from the East
> Who said it had horns though it was not a beast."

---

"Carmen Sylva," Queen of Roumania, makes good use of her literary talent. Next winter some of her dramatic works are to be produced in Vienna, and all the money is to go to the relief of pauper children.

